# BET bromodomain inhibitors suppress EWS-FLI1-dependent transcription and the IGF1 autocrine mechanism in Ewing sarcoma

**DOI:** 10.18632/oncotarget.9762

**Published:** 2016-06-01

**Authors:** Sudan N. Loganathan, Nan Tang, Jonathan T. Fleming, Yufang Ma, Yan Guo, Scott C. Borinstein, Chin Chiang, Jialiang Wang

**Affiliations:** ^1^ Department of Neuroscience and Pharmacology, Meharry Medical College, Nashville, TN, USA; ^2^ Department of Pharmacology, Vanderbilt University, Nashville, TN, USA; ^3^ Department of Neurosurgery, Union Hospital, Tongji Medical College, Huazhong University of Science and Technology, Wuhan, P.R. China; ^4^ Department of Cell and Developmental Biology, Vanderbilt University, Nashville, TN, USA; ^5^ Department of Neurological Surgery, Vanderbilt University, Nashville, TN, USA; ^6^ Department of Cancer Biology, Vanderbilt University, Nashville, TN, USA; ^7^ Department of Pediatrics, Vanderbilt University, Nashville, TN, USA

**Keywords:** BET bromodomain protein, Ewing sarcoma, EWS-FLI1, IGF1, BRD4

## Abstract

Ewing sarcoma is driven by characteristic chromosomal translocations between the *EWSR1* gene with genes encoding ETS family transcription factors (EWS-ETS), most commonly *FLI1*. However, direct pharmacological inhibition of transcription factors like EWS-FLI1 remains largely unsuccessful. Active gene transcription requires orchestrated actions of many epigenetic regulators, such as the bromodomain and extra-terminal domain (BET) family proteins. Emerging BET bromodomain inhibitors have exhibited promising antineoplastic activities via suppression of oncogenic transcription factors in various cancers. We reasoned that EWS-FLI1-mediated transcription activation might be susceptible to BET inhibition. In this study, we demonstrated that small molecule BET bromodomain inhibitors repressed EWS-FLI1-driven gene signatures and downregulated important target genes. However, expression of EWS-FLI1 was not significantly affected. Repression of autocrine IGF1 by BET inhibitors led to significant inhibition of the IGF1R/AKT pathway critical to Ewing sarcoma cell proliferation and survival. Consistently, BET inhibitors impaired viability and clonogenic survival of Ewing sarcoma cell lines and blocked EWS-FLI1-induced transformation of mouse NIH3T3 fibroblast cells. Selective depletion of individual BET genes partially phenocopied the actions of BET inhibitors. Finally, the prototypical BET inhibitor, JQ1, significantly repressed Ewing sarcoma xenograft tumor growth. These findings suggest therapeutic potential of BET inhibitors in Ewing sarcoma and highlight an emerging paradigm of using epigenetic agents to treat cancers driven by fusion transcription factors.

## INTRODUCTION

Ewing sarcoma is a group of highly malignant bone and soft tissue tumors that most often occur in children, teenagers, and young adults. It is defined by unique chromosomal translocations that give rise to fusion proteins comprising a RNA binding protein EWS (encoded by *EWSR1*) and one of the ETS family transcription factors [1]. EWS-FLI1 is the most common form (~85%) of EWS-ETS fusions. These chimeric proteins comprise the DNA-binding domain of ETS proteins and the amino-terminal region of EWS that functions as a strong transactivation domain. Additionally, inclusion of the constitutively active promoter of the *EWSR1* gene leads to high levels of expression [2]. As such, these chimeric transcription factors result in extensive transcriptional reprogramming in Ewing sarcoma cells [3]. Mounting evidence suggests that EWS-ETS fusions not only drive tumor initiation, but are also critically implicated in disease progression, underscoring the significance of these proteins as therapeutic targets for this disease [4]. However, EWS-ETS fusions do not contain structures readily recognized by small molecule compounds. Direct pharmacological intervention of EWS-ETS proteins remains largely unsuccessful. Further, no EWS-ETS target genes have been identified as effective stand-alone therapeutic targets.

Transcription is a complex process that involves orchestrated actions of many transcription factors, co-factors, RNA polymerase machineries and epigenetic regulators. Although it is often difficult to directly inhibit transcription factors, alternative pharmacological approaches, particularly agents selectively recognizing epigenetic regulators, have recently emerged to modulate oncogenic transcription programs [5]. Acetylated lysine residues on histone tails are marks of active transcription. Acetylated histone marks, such as H3K27ac, have profound implications in EWS-FLI1-driven transactivation [3]. Acetylated lysine residues can be recognized by highly conserved bromodomains that are found in more than 40 human proteins [6]. The BET family bromodomain proteins (include BRD2, BRD3, BRD4 and BRDT) are important readers for acetylated histones [6]. They contain two tandem bromodomains at the amino-terminus and play crucial roles in transcription activation and elongation. BRD4, the most extensively studied family member, is known to recruit the mediator complex that promotes transcription initiation [7, 8]. BRD4 also promotes transcription elongation by recruiting the positive transcription elongation factor b (P-TEFb), which releases promoter-proximal pausing of RNA polymerase II [9, 10]. While less well characterized, BRD2 and BRD3 appear to have similar functions in active gene expression [11]. Filippakopoulos and colleagues reported the first selective BET bromodomain inhibitor JQ1 in 2010 [12]. Shortly after discovery of JQ1, several groups independently demonstrated that inhibition of BET proteins suppressed expression and activity of MYC, a prominent oncogenic transcription factor that has long been deemed as “undruggable” [13–15]. These findings were followed by an explosion of studies demonstrating preclinical activities of BET bromodomain inhibitors in a wide range of human cancers [16–21]. The antineoplastic activities of BET inhibitors are often linked to their abilities to suppress oncogenic transcription factors, including MYC [13–15], MYCN [17], androgen receptor [19], GLI1/2 [20], and NF-κB [22]. The activity of BET inhibitors to attenuate aberrantly activated transcription provides an appealing strategy to indirectly target oncogenic transcription programs. It is reasonable to speculate that cancers driven by oncogenic transcription factors, such as Ewing sarcoma, may respond to BET bromodomain inhibitors. In this study, we demonstrate that Ewing sarcoma cells were highly sensitive to BET bromodomain inhibitors, JQ1 and i-BET762. Active transcription driven by EWS-FLI1 was significantly suppressed by BET inhibitors. JQ1 exhibited significant single agent activity in Ewing sarcoma xenograft models. These findings not only highlight the therapeutic potential of BET bromodomain inhibitors in this disease, but further support a paradigm of using epigenetic-based therapy to target oncogenic transcription programs in human cancers.

## RESULTS

### Inhibition of BET proteins represses global transcription driven by EWS-FLI1

**Figure 1:**
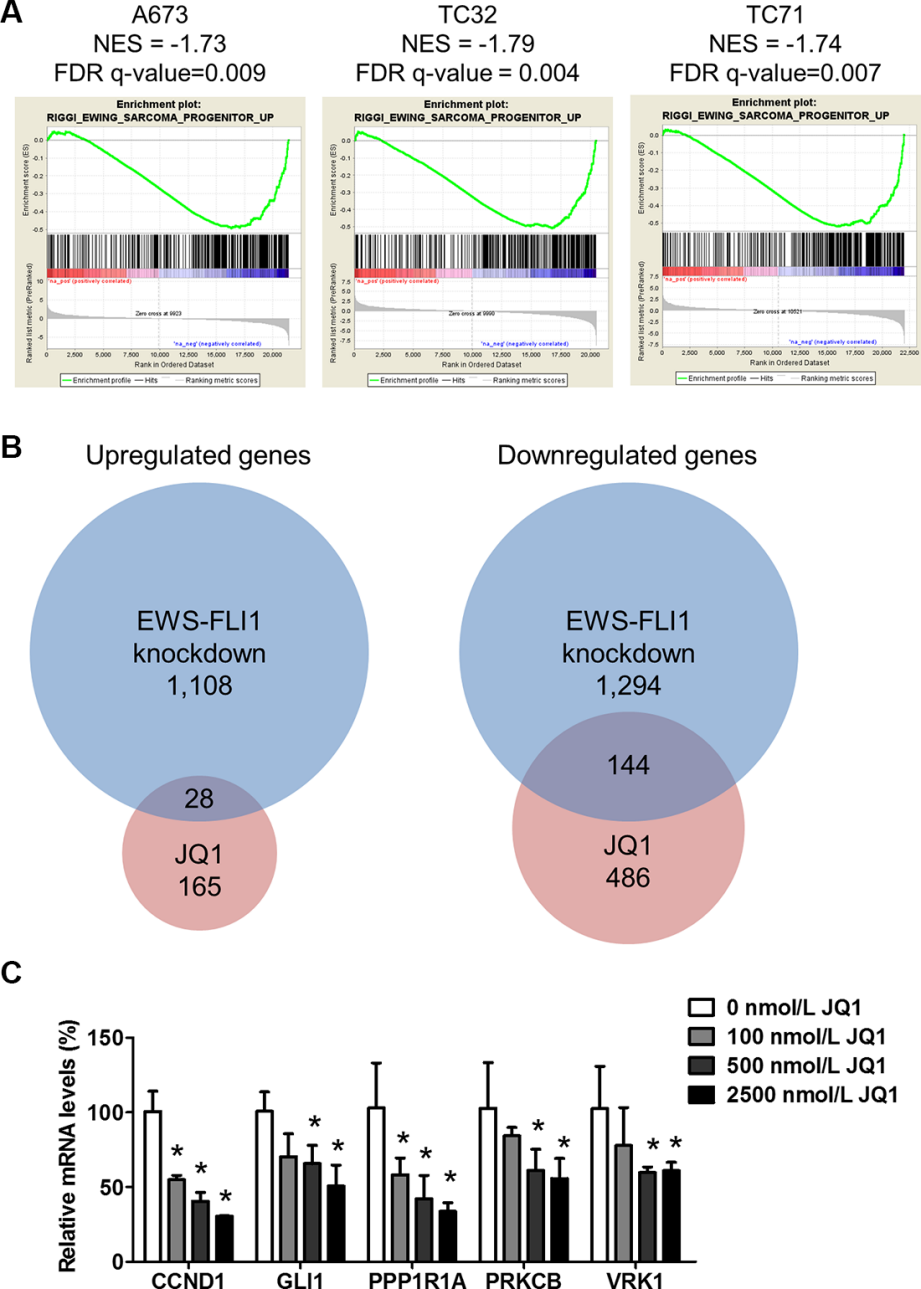
JQ1 suppresses EWS-FLI1-dependent transcription (**A**) Enrichment plots show that a EWS-FLI1-activated gene signature (Riggi_Ewing_Sarcoma_Progenitor_up, 430 genes) was suppressed in A673, TC32 and TC71 cells treated with 500 nmol/L JQ1 for 24 hours. FDR: false discovery rate. NES: Normalized enrichment score. (**B**) Venn diagrams demonstrate the overlap of genes changed more than 2 folds in JQ1-treated A673 cells and a published dataset in A673 cells upon EWS-FLI1 knockdown (GEO: GSE61950). (**C**) Expression of selected EWS-FLI1 target genes in TC32 cells treated with JQ1 at indicated concentrations for 24 hours. Actin was used as the loading control. In all figures, error bars represent standard deviations with the exception of Figure 7. All errors were calculated from at least three technical replicates. *p < 0.05 by Student's *t*-test compared with control samples.

EWS-FLI1 induces an oncogenic transcription program central to the molecular pathogenesis of Ewing sarcoma [23]. RNA interference-mediated depletion of EWS-FLI1 in Ewing sarcoma cells disrupts this transcription program, leading to differentiation, growth inhibition and cell death [1, 24]. On the contrary, introduction of EWS-FLI1 transforms mouse or human mesenchymal progenitor cells, which are putative cell of origin for Ewing sarcoma, and generates expression patterns that resemble Ewing sarcoma cells [25–27]. We first examined the impact of BET inhibition on expression profiles of Ewing sarcoma cells by RNA-seq. Transcriptomes of three Ewing sarcoma cells lines, A673, TC32 and TC71, were analyzed following treatment of 500 nmol/L JQ1 for 24 hours. Gene set enrichment analysis (GSEA) was employed to assess the changes in EWS-FLI1-regulated transcription modules. In all three tested lines, JQ1 significantly suppressed a gene signature that was upregulated by EWS-FLI1 when expressed in human mesenchymal progenitor cells [27] (Figure 1A), suggesting that BET proteins play important roles to sustain the EWS-FLI1-dependent transcription program. We also compared changes in global gene expression following JQ1 treatment to a published dataset that analyzed the impact of EWS-FLI1 knockdown on transcriptome, both in A673 cells [3] (Figure 1B). We found that a substantial percentage (~22%) of genes downregulated > 2 folds upon JQ1 treatment were also repressed by knockdown of EWS-FLI1. Conversely, while knockdown of EWS-FLI1 induced over 1000 genes by at least 2 folds, JQ1 upregulated 293 genes, of which only 28 overlapped with the group induced by EWS-FLI1 knockdown (Figure 1B). These results were consistent with the primary functions of BET proteins in transcription activation. While compared with several chemo drugs reported to interfere with the transcriptional activity of EWS-FLI1, such as mithramycin [28] and cytarabine [29], very limited overlap was identified (Supplementary Figure 1). These results suggest that inhibition of BET proteins selectively targets expression of a subset of genes that are upregulated by EWS-FLI1.

Because BET proteins are readers for histone acetylation, it is reasonable to speculate that EWS-FLI1 target genes sensitive to BET inhibition may be associated with high acetylation in adjacent chromatin region. Riggi and colleagues recently published a study that detailed the global impact of EWS-FLI1 on chromatin remodeling and gene expression [3]. EWS-FLI1-bound chromatin is associated with heterogeneous levels of H3K27 acetylation, which is a mark of enhancers and recognized by BRD4 [30]. Combining RNA-seq data and H3K27ac ChIP-seq results, Riggi and colleagues identified genes that are directly repressed or activated by EWS-FLI1 through histone acetylation reprogramming [3]. When EWS-FLI1 was depleted, these target genes were significantly activated or repressed with coordinated changes in the levels of adjacent H3K27ac. Hence, we validated the transcriptome analysis using EWS-FLI1 target genes selected from the top-ranked gene list of the Riggi study, including *CCND1*, *PPP1R1A*, *PRKCB* and *VRK1* [3]. Expression of these genes was decreased in a concentration-dependent manner following exposure to JQ1 in TC32 cells (Figure 1B). We also found that JQ1 significantly downregulated expression of *GLI1* in Ewing sarcoma cells (Figure 1B). *GLI1* is a direct EWS-FLI1 target gene and a key regulator of the EWS-FLI1-dependent transcriptional network that drives tumorigenesis in Ewing sarcoma [31–33]. In addition, *GLI1* was recently found as a direct BRD4 target gene [20]. Similar changes in expression of these EWS-FLI1 target genes were observed in additional Ewing sarcoma cells, such as TC71 (Supplementary Figure 2A). Also, administration of i-BET762 (GSK525762), a BET bromodomain inhibitor in phase I trial, imposed similar impact on expression of EWS-FLI1 target genes (Supplementary Figure 2B). However, *MYC*, an extensively documented BRD4 target gene, was not downregulated upon BET inhibition (Supplementary Figure 2C), despite earlier studies suggest that *MYC* transcription may be regulated by EWS-FLI1 through indirect mechanisms [34, 35]. In addition to these established EWS-FLI1 target genes, we also found that BET inhibition resulted in upregulation of pro-apoptotic genes, such as *BIM*, and downregulation of anti-apoptotic genes, such as *BCL2* and *BIRC3* (also known as *cIAP2*) (Supplementary Figure 2D). Collectively, these results suggest that BET proteins are crucially implicated in transcription of EWS-FLI1 target genes.

### EWS-FLI1 expression is not sensitive to BET inhibitors

**Figure 2:**
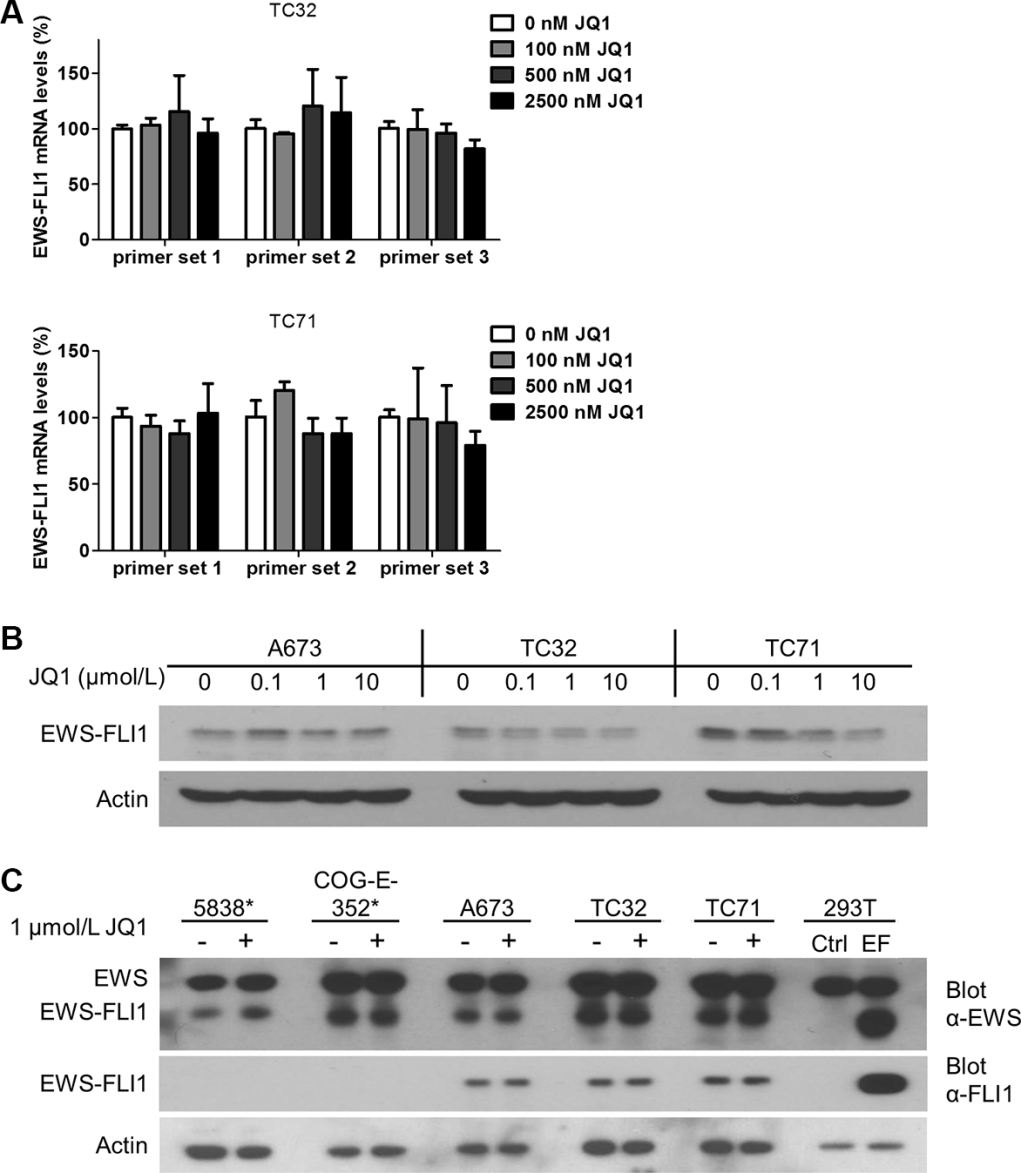
EWS-FLI1 expression is not affected by BET inhibition (**A**) EWS-FLI1 mRNA and (**B**) protein levels in A673, TC32 and TC71 cells treated with JQ1 at indicated concentrations for 24 hours. (**C**) EWS-FLI1 immunoblotting in Ewing sarcoma cell lines treated with 1 μmol/L JQ1 for 24 hours. *: 5838 and COG-E-352 express EWS-ERG, thus serving as a negative control for EWS-FLI1. 293T cells were transfected with pCDH-puro-EWS-FLI1 (EF) to produce recombinant EWS-FLI1 protein as the positive control. Ctrl.: vector only.

Two groups recently reported that JQ1 suppressed EWS-FLI1-mediated transcription through downregulating EWS-FLI1 at both mRNA and protein levels [36, 37]. One of these two studies also showed that BRD4 bound to the promoter regions of *EWSR1* [37], which regulates transcription of both the wild type and the fusion genes. To test this possibility, we employed quantitative real-time PCR (qRT-PCR) to measure EWS-FLI1 mRNA levels following JQ1 treatment using three distinct pairs of primers that span the junction region of EWS-FLI1 fusion region [38], including the primer set used in the Hensel study (Supplementary Table 1). However, no significant downregulation of EWS-FLI1 mRNA levels were observed in both TC32 and TC71 cells following JQ1 treatment across a broad range of concentrations (Figure 2A). We further examined EWS-FLI1 protein levels using an antibody specifically recognizing the carboxyl-terminal region of FLI1 and detected no significant changes in the presence of JQ1 in all tested lines, including those used in the two published studies (Figure 2B). Although a minor decrease of EWS-FLI1 in TC71 cells treated with 10 μmol/L JQ1 could be detected, this change could be secondary to toxicity of JQ1 at high concentrations (Figure 2B). Further, an anti-EWS antibody specific to the amino-terminus region of EWS-FLI1 was also employed to validate the results. Two Ewing sarcoma cell lines expressing EWS-ERG were included to compare with the EWS-FLI1-expressing lines. Consistently, neither EWS-ERG nor EWS-FLI1 was significantly downregulated by JQ1 (Figure 2C). Also, levels of wild type EWS were not significantly altered by JQ1 (Figure 2C). These data suggest that a direct impact on transcription of EWS-FLI1 is unlikely a key mechanism mediating the functional crosstalk between BET proteins and EWS-FLI1.

### Expression of EWS-FLI1 target genes rapidly decreases following BET inhibition

**Figure 3:**
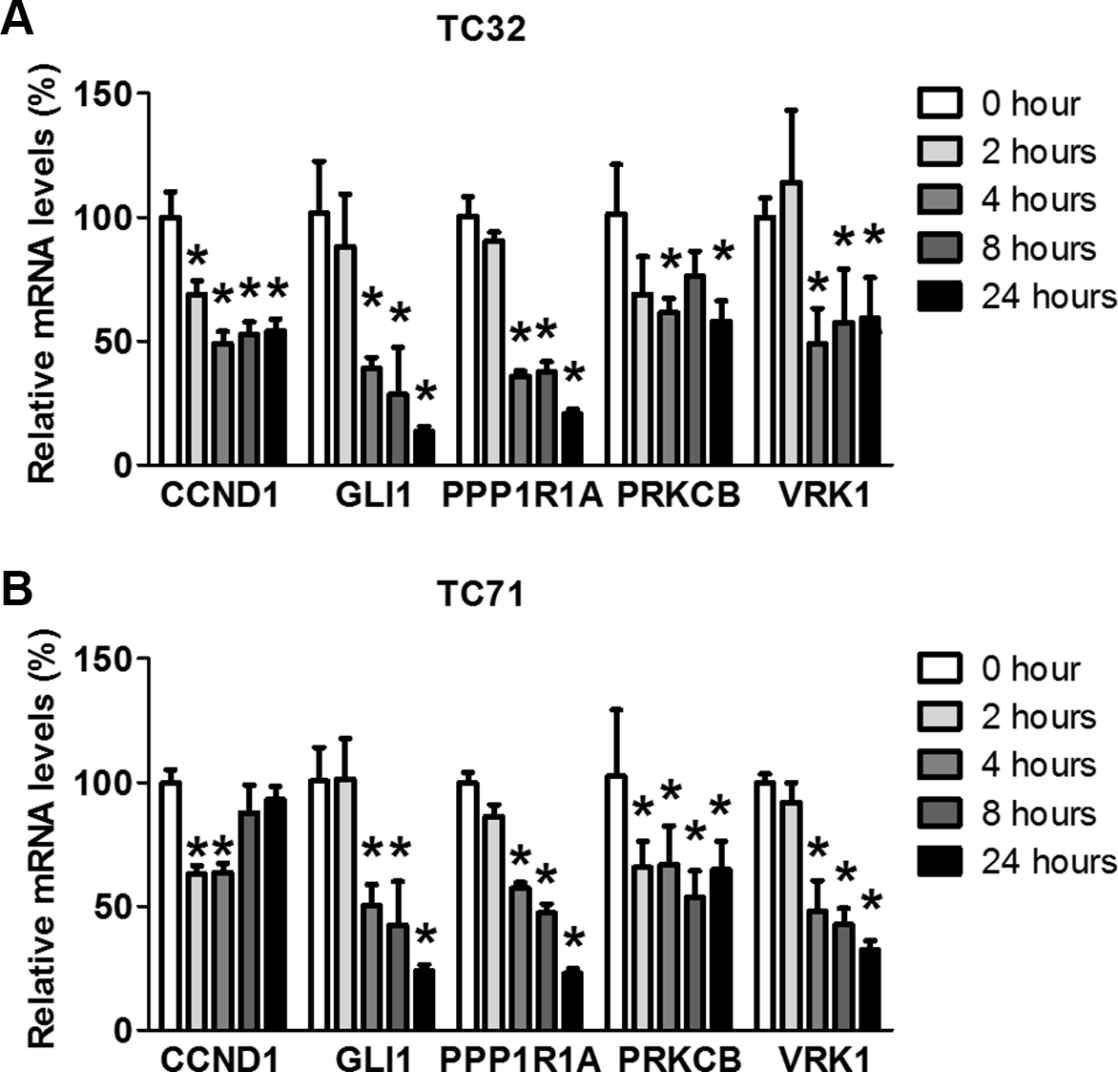
JQ1 rapidly decreases expression of EWS-FLI1 target genes (**A**) TC32 or (**B**) TC71 cells were treated with 500 nmol/L JQ1 for 2, 4, 8 or 24 hours. Expression of selected EWS-FLI1 target genes was shown by qRT-PCR. *p < 0.05 by Student's *t*-test compared with control samples.

The impact of BET inhibitors on EWS-FLI1-dependent transcription may involve direct effects or indirect effects mediated via BET inhibition-induced growth arrest and cell death. Genes directly regulated by BET proteins are expected to exhibit rapid changes following JQ1 administration, while genes affected through indirect effects may change after a substantial delay. As such, we measured the time-dependent impact of JQ1 on expression of selected EWS-FLI1 genes. Our results showed that downregulation of some genes, such as *CCND1* and *PRKCB*, were obvious as early as 2 hours after addition of JQ1. At 4 hours post JQ1 treatment, essentially all tested genes were significantly suppressed (Figure 3A and 3B), suggesting that the effects of BET inhibition on transcription of EWS-FLI1 target genes likely involve direct regulation on chromatin. Of note, in TC71 cells, *CCND1* mRNA levels were restored following the initial decrease (Figure 3B), suggesting that indirect feedbacks may attenuate the impact of BET inhibition on expression of EWS-FLI1 target genes. Additional EWS-FLI1-activated genes were significantly suppressed 4 hours after JQ1 treatment, such as *CER1*, *CYPF22*, *DCDC2*, and *RNF182* (Supplementary Figure 3A). Conversely, genes repressed by EWS-FLI1, such as *ERFF1*, *CABLES1*, and *TGBI*, were not increased (Supplementary Figure 3B). Their expression levels after 24-hour exposure to JQ1 remained largely steady (Supplementary Figure 3C and 3D). Furthermore, doxorubicin, a chemo drug commonly used in the management of Ewing sarcoma, did not suppress expression of selected EWS-FLI1 target genes (Supplementary Figure 3E). Hence, the effects of BET inhibitors on EWS-FLI1-driven transcription are unlikely mediated through indirect impact of BET inhibition on cell growth and survival.

### Depletion of individual BET genes partially phenocopies BET inhibitors

**Figure 4:**
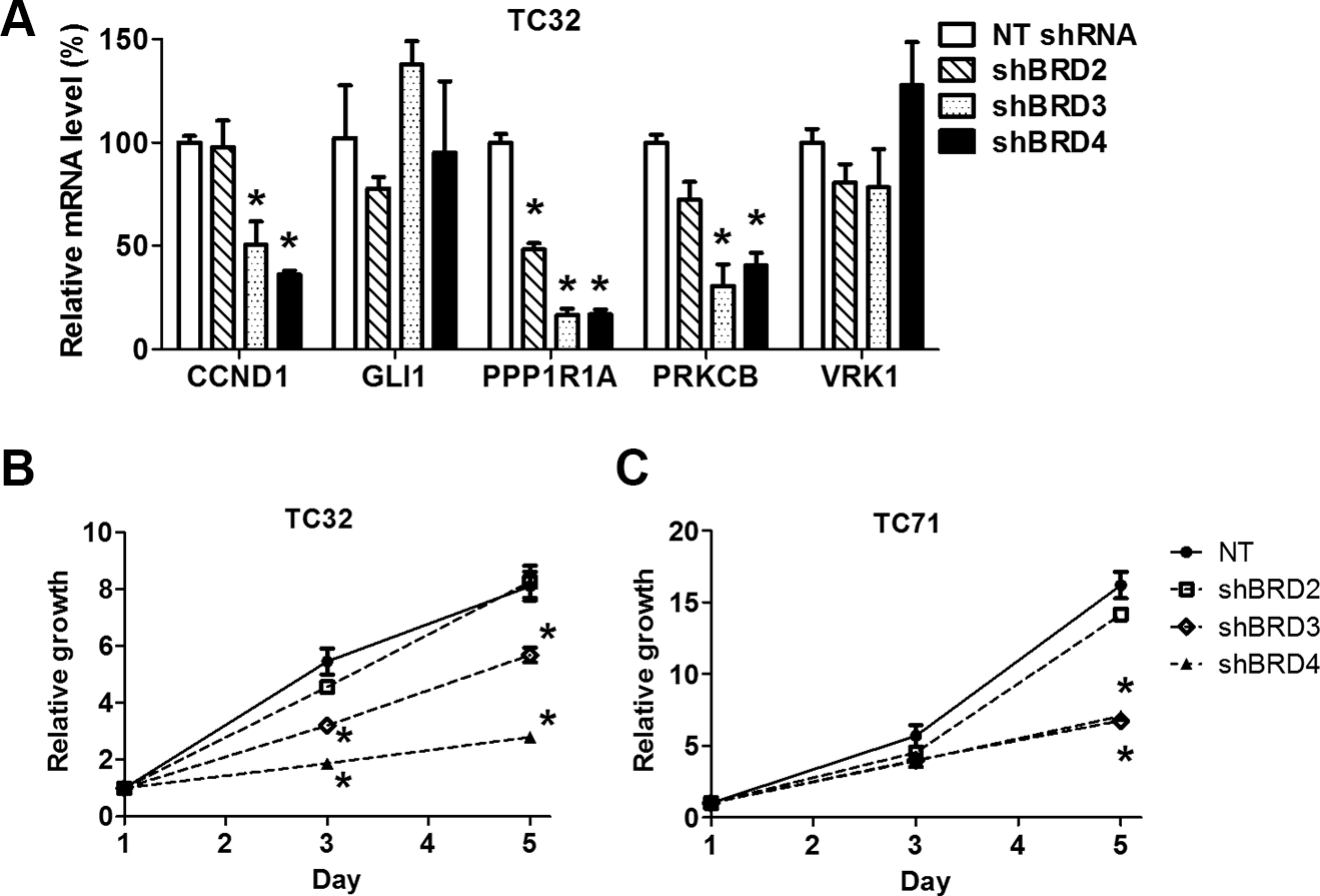
Depletion of individual BET proteins represses expression of EWS-FLI1 target genes and impairs cell growth (**A**) TC32 cells were infected with lentivirus directing expression of a non-targeting (NT) shRNA or shRNA selectively targeting BET genes. Expression of selected EWS-FLI1 target genes was assessed by qRT-PCR. (**B**) TC32 or (**C**) TC71 cell growth rates were calculated by normalizing cell titer readings to the mean values of the corresponding groups on day 1, which was assigned a value of 1. *p < 0.05 by unpaired Student's *t*-test compared with values of the NT group of the same day.

To further validate the specificity of the activities of BET inhibitors, we selectively depleted BET proteins using lentivirus-mediated expression of shRNA specific to individual BET genes as described in our previous study (Supplementary Figure 4A) [16]. Although most previous studies attribute the activities of BET inhibitors to inhibition of BRD4, our results showed that knockdown of either BRD3 or BRD4 partially recapitulated the ability of BET inhibitors to downregulate EWS-FLI1 target genes (Figure 4A and Supplementary Figure 4B). However, the impact of BRD2 depletion appeared to be less significant compared with knockdown of the other two BET family members. Additionally, depletion of BRD3 or BRD4 significantly reduced the growth rate of TC32 or TC71 cells, while BRD2 knockdown did not (Figure 4B and 4C). These findings suggest that full activation of EWS-FLI1-driven transcription program may require at least both BRD3 and BRD4.

### BET inhibition impairs the insulin-like growth factor 1 (IGF1) autocrine mechanism

**Figure 5:**
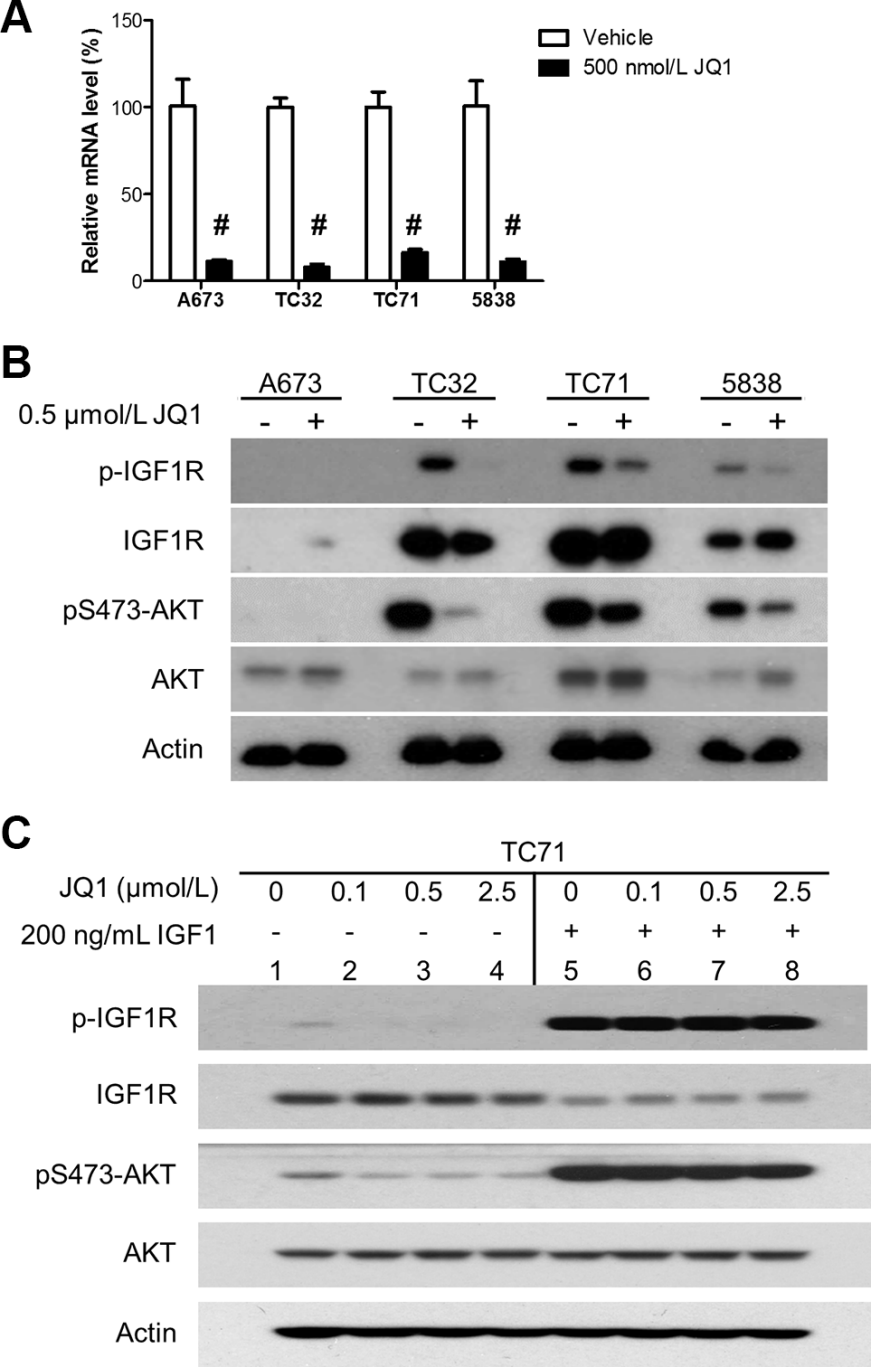
BET inhibition impairs the IGF1 autocrine mechanism (**A**) A673, TC32, TC71 and 5838 cells were treated with 500 nmol/L JQ1 for 24 hours. IGF1 mRNA levels were determined by qRT-PCR. ^#^*p* < 0.001 by Student's *t*-test compared with the control groups. (**B**) Cells were treated as described above and subject to immunoblotting for indicated proteins. (**C**) TC71 cells were treated with JQ1 at indicated concentrations for 24 hours and stimulated with 200 ng/mL human recombinant IGF1 for 1 hour. Cells were then lysed and subject to immunoblotting.

It has been extensively documented that the IGF1R pathway is frequently activated in Ewing sarcoma and has important proliferative and prosurvival functions [39, 40]. Anti-IGF1R therapies have exhibited promising preclinical activities in Ewing sarcoma models, but appear to be inadequate to induce sustained clinical response either as monotherapy or in combination with mTOR inhibitors, in part due to toxicity of blocking IGF1R signaling in normal tissues [41–43]. Activation of the IGF1R pathway in Ewing sarcoma is critically sustained by IGF1 produced by tumor cells [44]. Because IGF1 is a direct target gene of EWS-FLI1, this autocrine loop is largely driven by EWS-FLI1 in Ewing sarcoma [45]. Additionally, IGF1 is among a small subset of genes commonly activated by EWS-FLI1 and other Ewing sarcoma fusion proteins, such as EWS-ERG, underscoring its significance in this disease [45]. In the expression profiling assays, we found that IGF1 expression was highly sensitive to JQ1 in all three lines that we have tested. These observations were confirmed by qRT-PCR (Figure 5A). In addition, JQ1 significantly decreased phosphorylation of IGF1R and AKT in multiple Ewing sarcoma cell lines, including 5838 that expresses EWS-ERG (Figure 5B). The A673 line was an exception, because it expressed very low levels of IGF1R, thus having low AKT activity that was irresponsive to JQ1 (Figure 5B). Recombinant IGF1 rescued inhibition of the IGF1R/AKT pathway by JQ1 (Figure 5C). Hence, BET proteins are essential to sustain the IGF1 autocrine mechanism in Ewing sarcoma, suggesting an alternative approach to block this important pathway in tumors without perturbation of IGF1 signaling in normal tissues.

### BET inhibitors compromise proliferation and survival of Ewing sarcoma cells

**Figure 6:**
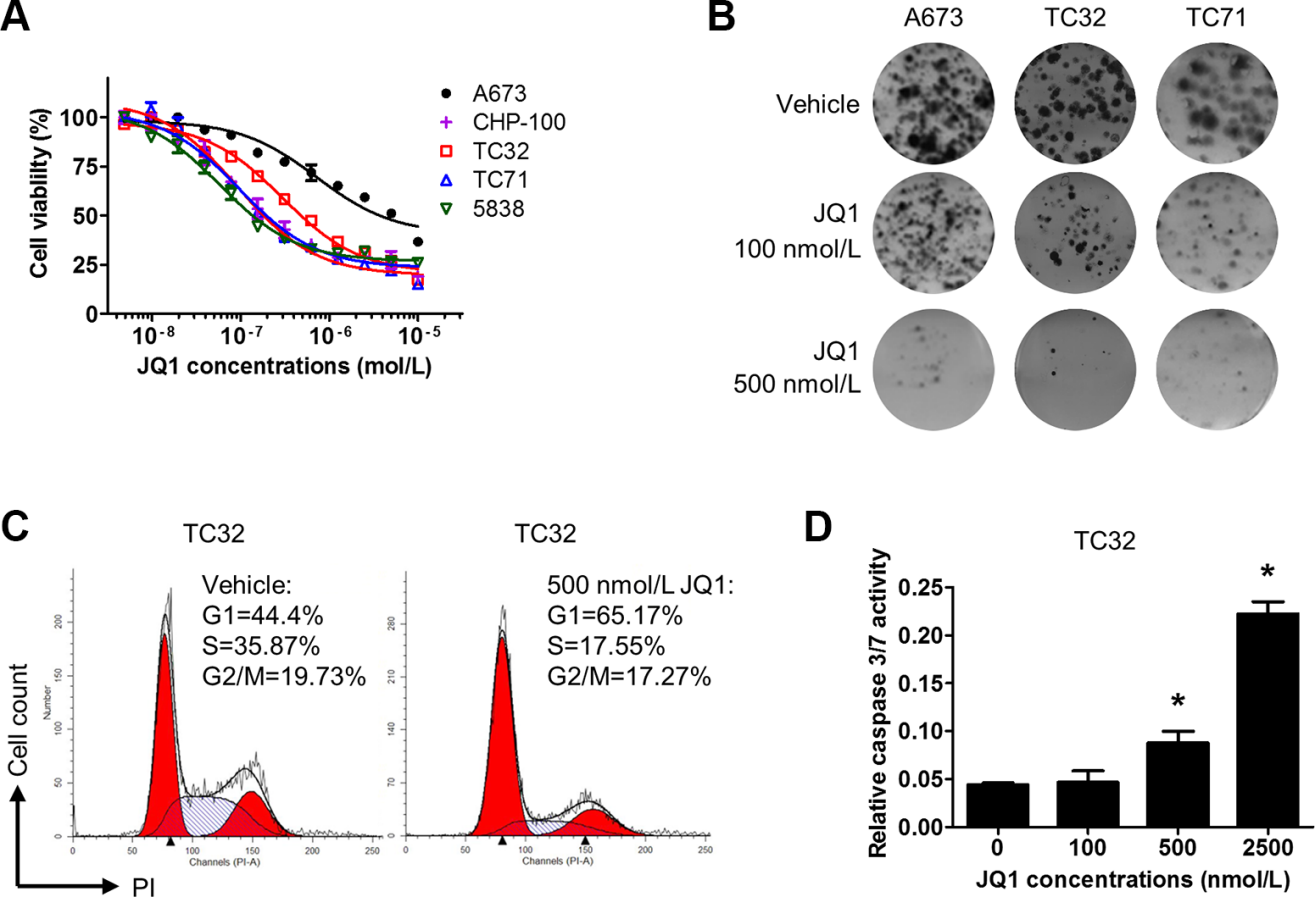
BET inhibition compromises proliferation and survival of Ewing sarcoma cells (**A**) Dose-response curves for JQ1 in Ewing sarcoma cells following a 5-day treatment. (**B**) 2,000 A673, CHP100, or TC32 cells were treated with 100 or 500 nmol/L JQ1 for 5 days. After drug withdrawal, cells were incubated for 7 more days prior to staining with 0.5% crystal violet. Representative images are shown. (**C**) TC32 cells were treated with 500 nmol/L JQ1 for 24 hours and fixed for propidium iodide staining. Cell cycle distribution was calculated by the ModFit software. (**D**) TC32 cells were treated with JQ1 at indicated concentrations for 3 days. Caspase 3/7 activity was determined by the Caspase3/7-Glo kit (Promega) and normalized to cell titer readings of the corresponding groups.

We next examined how inhibition of BET proteins might affect proliferation and survival of Ewing sarcoma cells. In all tested Ewing sarcoma cell lines, we showed that JQ1 and i-BET762 decreased cell viability in a concentration-dependent manner (Figure 6A and Supplementary Figure 5A). The concentrations required to reduce cell viability by 50% (IC_50_) were under 100 nmol/L for CHP100, TC71 and 5838 cells, approximately 200 nmol/L for TC32 and 700 nmol/L for A673. It is worth noting that the IGF1R-low line A673 had the highest IC50 value among these lines, thus supporting the hypothesis that the IGF1 autocrine loop is a key target of BET inhibitors in Ewing sarcoma. In addition, we transiently treated Ewing sarcoma cells with JQ1 and let them to develop colonies after drug withdrawal. These experiments showed that transient exposure to 500 nmol/L JQ1 was sufficient to block the majority of colony formation capacity in Ewing sarcoma lines (Figure 6B), indicating that BET inhibition imposes prolonged damage to tumorigenicity of Ewing sarcoma cells. Further, JQ1 treatment modestly reduced the percentage of cells in S phase and induced concentration-dependent activation of caspase 3 (Figure 6C and 6D). To further assess the impact of BET inhibition on the core malignant programs of Ewing sarcoma cells, we introduced lentivirus-mediated stable expression of EWS-FLI1 into mouse NIH3T3 cells. NIH3T3 cells are susceptible to EWS-FLI1 and may grow in soft agar once transformed by EWS-FLI1 [46]. Our data showed that exposure to JQ1 at 100 or 500 nmol/L significantly impaired anchorage independent growth of EWS-FLI1-expressing NIH3T3 cells (Supplementary Figure 5B). Taken together, these data suggest that BET inhibition compromises the core malignant features of Ewing sarcoma cells in part through induction of growth arrest and apoptosis.

### JQ1 represses Ewing sarcoma xenograft tumor growth

**Figure 7:**
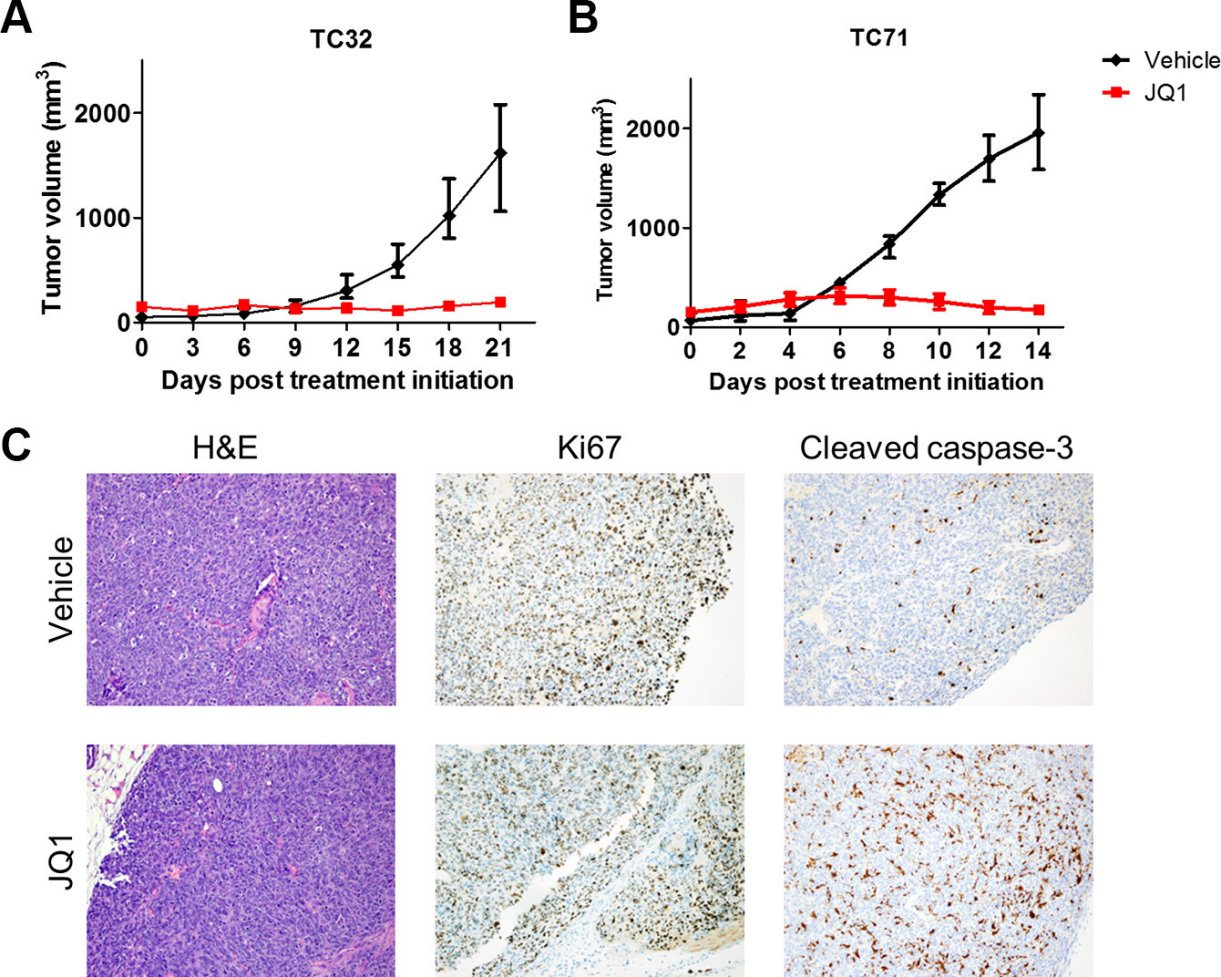
JQ1 impairs Ewing sarcoma xenograft tumor growth (**A**) Athymic nude mice bearing TC32 (*n* = 8) or (**B**) TC71 (*n* = 5) flank xenograft tumors were treated with 50 mg/kg JQ1 twice per day. Data presented are median tumor size. Error bars represent interquartile range. (**C**) TC32 tumors treated with JQ1 for 3 weeks were dissected and stained using hematoxylin and eosin, or antibodies specific to Ki67 or cleaved caspase-3. Representative images (200×) are presented.

Next, the *in vivo* therapeutic potential of BET inhibitors was examined in subcutaneous xenograft models of Ewing sarcoma. In both TC32 and TC71 xenograft models, tumor growth was either halted or marginally increased following administration of JQ1, whereas tumors in the control arm rapidly grew (Figure 7A and 7B). T32 xenograft tumors were resected and stained for the proliferation marker, Ki67, and an apoptosis marker, cleaved caspase 3 (Figure 7C). JQ1-treated TC32 tumors did not show significant difference in Ki67-positive staining compared with control tumors. However, cleaved caspase 3 staining was significantly enhanced in JQ1-treated tumors, suggesting that increased cell death may play a key role in mediating the *in vivo* activities of BET inhibitors against Ewing sarcoma.

## DISCUSSION

The oncogene addiction concept suggests that some cancers are crucially dependent on the products of one or a few aberrantly activated oncogenes [47]. This model has thus far been the most successful rationale for development of targeted anti-cancer therapies, exemplified by imatinib in BCR-ABL-driven chronic myeloid leukemia. It has been extensively recognized that Ewing sarcoma also follows the oncogene addiction model. However, the challenges to develop pharmacological agents for transcription factors, like EWS-FLI1, severely impede translation of this knowledge to clinic success. Several alternative strategies have recently been reported. Certain chemotherapy drugs, such as cytarabine, mithramycin and trabectedin, have been shown to impair the transcriptional activity of EWS-FLI1 [28, 29, 48, 49]. However, the therapeutic indexes of these chemo drugs are limited by their toxicity, which may be overcome by further modifications of their chemical structures [50]. In addition, a first-in-class compound selectively targeting the interaction between EWS-FLI1 and RNA helicase A (YK-4-279) has been developed to modulate EWS-FLI1 activities [51]. Therapeutic potential of this novel strategy remains to be examined in clinics.

Epigenetics plays essential roles in regulating the activity and specificity of transcription. Over the past five years, small molecule compounds have been developed to target a growing number of epigenetic regulators, creating many new opportunities to modulate oncogenic transcription factors in cancers [5]. For example, the lysine demethylase LSD1 (also known as KDMA1) was recently identified as an important epigenetic partner of EWS-FLI1 and LSD1 inhibitors showed promising activities in preclinical Ewing sarcoma models [52, 53]. The BET family epigenetic readers have profound interactions with several oncogenic transcription factors [8]. In the current study, we demonstrated that genetic or pharmacological targeting of BET proteins attenuated EWS-FLI1-mediated transcription activation. Consequently, BET inhibitors exhibited single-agent activity against Ewing sarcoma *in vitro* and *in vivo*. Ewing sarcoma in general appeared to be highly sensitive to BET inhibitors. Several Ewing sarcoma cell lines showed IC50 values below 100 nmol/L for JQ1. Transient exposure to JQ1 at concentrations in the submicromolar range dramatically impaired clonogenic survival of Ewing sarcoma cells, suggesting that BET inhibition results in prolonged damage to tumorigenicity of Ewing sarcoma cells. Further, BET inhibition undermined EWS-FLI1 induced transformation of mouse NIH3T3 cells, as shown by prohibition of anchorage-independent growth in soft agar. Consistently, administration of JQ1 essentially halted growth in TC32 or TC71 Ewing sarcoma xenograft tumors. BET inhibition induced significant caspase activation in xenograft tumors but did not reduce Ki67-positive cells. One possible explanation is that the half-life of JQ1 is short *in vivo* [54], so that some cells did not permanently exit cell cycle and remained Ki67 positive. Further studies to understand the *in vivo* therapeutic activities of BET inhibitors in Ewing sarcoma models are warranted. These observations collectively support an essential role of BET proteins in the maintenance of a core oncogenic network in Ewing sarcoma. Many other tumors sensitive to BET inhibitors are also driven by aberrantly activated oncogenic transcription factors. On the contrary, tumors initiated by other mechanisms, such as ERBB2-driven breast cancer [55], pancreatic cancer [56], and glioblastoma [16], appear to be less susceptible to BET inhibitors as monotherapy. Hence, our findings and mounting evidence suggest a model that BET inhibitors have particularly important therapeutic indications in cancers driven by amplified or mutated transcription factors.

The mechanisms through which EWS-FLI1 establishes the unique gene expression pattern in Ewing sarcoma have recently emerged. The Riggi study and results from other groups show that EWS-FLI1 preferentially binds to chromatin regions enriched for GGAA repeats, induces formation of *de novo* enhancers, and activate transcription of target genes [3, 57]. EWS-FLI1-binding sites are universally associated with H3K4me3, which is a mark of transcription initiation. However, H3K27ac levels, a mark of enhancer activity, are heterogeneous at EWS-FLI1 binding. The Riggi study unveiled a strong correlation between changes in EWS-FLI1 target gene expression and changes in proximal H3K27ac levels, suggesting that histone acetylation and enhancer activation is critical to transcription activation driven by EWS-FLI1 [3]. BET proteins bind to H3K27ac in addition to other acetylated lysine residues and have important roles in regulation of enhancer activity [30, 58]. Our results showed that BET inhibition downregulated top-ranked EWS-FLI1-activated genes that are associated with high levels of H3K27ac in adjacent chromatin regions, but did not upregulate EWS-FLI1-repressed genes associated with low levels of H3K27ac. These observations agree with the selective roles of BET proteins in promoting active transcription. The mechanisms mediating the crosstalk between BET proteins and EWS-FLI1 remain to be fully elucidated. Nevertheless, the impact of BET inhibitors on transcriptome of Ewing sarcoma appeared to be distinct from those of chemo drugs, such as doxorubicin, mithramycin and cytarabine, supporting a specific role of BET protein in EWS-FLI1 regulation. However, BET inhibition showed limited impact on EWS-FLI1-mediated transcriptional repression, which is increasingly recognized as a key driver in oncogenesis of Ewing sarcoma [52]. As such, the ability of BET inhibitors to modulate EWS-FLI1 has its limitation, and combinations with additional epigenetic agents targeting EWS-FLI1-mediated transcriptional repression may improve the outcomes. Collectively, our findings suggest that the crosstalk between BET proteins and EWS-FLI1 is potentially mediated via adjacent histone acetylation marks and enhancer activities. A comprehensive ChIP-seq study is necessary to provide further mechanistic insights by comparing global distribution of EWS-FL11 and BET proteins relative to key histone acetylation marks in Ewing sarcoma. Other regulatory mechanisms may exist. Recent studies reported significant downregulation of EWS-FLI1 at both mRNA and protein levels upon inhibition of BET proteins [36, 37]. In contradiction to these studies, our group did not recapitulate these observations. Further, BET inhibition did not upregulate genes repressed by EWS-FLI1 as assessed by GSEA (data not shown) or qRT-PCR, suggesting that some of the functions of EWS-FLI1 remain intact. Additional independent studies are needed to further test whether BET proteins directly affect expression of EWS-FLI1.

IGF1 is an important transcriptional target of EWS-FLI1 [45]. The resultant IGF1 autocrine loop is essential to sustain adequate activation of the IGF1R/PI3K/AKT signaling pathway in Ewing sarcoma [40]. IGF1R kinase inhibitors and neutralizing antibodies have shown significant preclinical activities in Ewing sarcoma models [40]. Anti-IGF1R therapy has shown efficacy in a small subset of Ewing sarcoma patients. However, difficulty in selecting the sensitive population and toxicity associated with prolonged inhibition of IGF1R signaling in normal tissues impede clinical application of this therapy [41–43]. The potent and universal blockade of IGF1 expression by BET inhibitors in Ewing sarcoma was unexpected. IGF1 is among the most sensitive genes to BET inhibitors. Also, the link between IGF1 expression and BET proteins appears to be unique to Ewing sarcoma (unpublished results), which is consistent with the unique ability of EWS-FLI1 to stimulate IGF1 expression in Ewing sarcoma [45]. We also noticed that Ewing sarcoma cell lines expressing low levels of IGF1R, such as A673, were less sensitive to JQ1 in comparison to IGF1R-overexpressing lines, underscoring the significance of the IGF1 autocrine mechanism as a key target of BET inhibitors in Ewing sarcoma. It is an appealing strategy using BET inhibitors to block the IGF1 autocrine mechanism in Ewing sarcoma patients with high IGF1R expression, which is expected to spare normal tissues from toxicity of anti-IGF1R therapy. Hence, future studies are warranted to determine whether IGF1R-overexpressing Ewing sarcomas are more sensitive to BET inhibitors than Ewing sarcomas independent on the IGF1R/AKT pathway.

In summary, our study identifies a significant role of the BET family epigenetic readers in regulation of the transcriptional activity of EWS-FLI1. These findings suggest a novel epigenetic-based treatment for Ewing sarcoma, particularly those resistant to currently available chemotherapies. Our results also suggest a therapeutic paradigm that tumors driven by aberrantly activated oncogenic transcription factors are preferential targets for BET inhibitors. This strategy can be readily extended to other cancer types initiated by fusion transcription factors.

## MATERIALS AND METHODS

### Cell culture

Ewing sarcoma cell lines were generous gifts from Patrick Grohar at Van Andel Research Institute. COG-E-352 was a gift from the Children's Oncology Group Cell Culture/Xenograft Repository. Cells were maintained at 37^°^C in 5% CO_2_ with RPMI-1640 medium for TC32, TC71, 5838, CHP-100 and COG-E-352 cells, and DMEM for A673 and mouse NIH3T3 cells. All cultures were supplemented with 10% fetal bovine serum and 100 U/mL penicillin-streptomycin. Tissue culture reagents were purchased from Life Technologies. Identity of Ewing sarcoma cell lines used in this study were checked by PCR for expression of EWS-FLI1.

### Immunoblotting assays

Cell lysates were made using RIPA buffer (Sigma) supplemented with a cocktail of protease inhibitors and phosphatase inhibitors (Sigma) at 4^°^C. The rabbit polyclonal antibody specific to the carboxyl-terminal region of FLI1 (sc-356) and the mouse monoclonal antibody targeting the N-terminus of EWS (sc-48404) were purchased from Santa Cruz Biotechnology. Other antibodies used in this study are described in our recent publications [59].

### Plasmids and lentivirus production

The pLKO.1 lentiviral vectors encoding the non-targeting shRNA or shRNA sequences specific to BRD2 (TRCN000006310), BRD3 (TRCN0000021376), and BRD4 (TRCN0000196576) have been described in our previous publications [16]. The cDNA sequence of EWS-FLI1 was subcloned into the NheI and NotI sites of the lentiviral vector pCDH-CMV-MCS-EF1-Puro. Lentivirus was produced by co-transfection of the lentiviral vectors with the packaging vectors psPAX2 and pCl-VSVG (Addgene) into 293FT cells. Cells were infected by viral supernatant at an approximate MOI of 5. Cells were selected with 1 μg/mL puromycin for at least 48 hours prior to experimentation.

### Quantitative real-time PCR

Total RNA was isolated using the GE Healthcare Illustra RNAspin kit with on-column DNAase treatment and reverse transcribed using the Bio-Rad iScript cDNA synthesis kit. Universal SYBR-Green Mastermix (Bio-Rad) was used for RT-PCR. The reaction comprises of 40 cycles at 95°C for 20 seconds and 60°C for 45 seconds. Primers are listed in Supplementary Table 1. Beta-Actin was used as the loading control.

### RNA-seq and gene set enrichment analysis

A673, TC32, and TC71 cells were treated with DMSO or 500 nmol/L JQ1 for 24 hours. Total RNA was extracted as described above. RNAseq were performed by Vanderbilt Technologies for Advanced Genomics (VANTAGE). The Illumina TruSeq RNA Sample Preparation kit was used for library preparation. The RNAseq data went through multiple stages of thorough quality control as recommended [60]. Raw data and alignment quality control were performed using QC3, and gene quantification quality control was conducted using MultiRankSeq. Raw data were aligned with TopHat2 against human transcript genome HG19. Gene expression was quantified using Cufflinks. Differentially expression analysis is performed using Cuffdiff command from Cufflinks package.

The GSEA method (http://www.broadinstitute.org/gsea) was employed for functional analysis [61]. This approach determines whether an a priori defined set of genes shows statistically significant differences between two phenotypes. The complete list of genes and their scores were used in GSEA with a focus on the C2 curated gene sets (CGP collection, chemical and genetic perturbations). According to the developer's instructions, the false discovery rate (FDR) q value represents ‘the estimated probability that a gene set with a given normalized enrichment score represents a false positive finding’.

### Cell viability assay

Ewing sarcoma cells were plated at 2000 cells/well in 96-well plates and treated with BET inhibitors following a 2-fold serial dilution. Five days later, cell viability was measured using the Sensolyte Cell Viability Assay kit (Anaspec). The dose-response curves and IC_50_ values were determined using GraphPad Prism 5 following a nonlinear regression (three parameters, least squares fit) method.

### Cell cycle analysis

Cells were treated for 24 hours with either DMSO or 500 nmol/L JQ1, fixed with 75% ethanol and stained by 10 μg/mL propidium iodide in the presence of 100 μg/mL RNase. Propidium iodide staining was measured by flow cytometry on a BD LSRFortessa Cell Analyzer. Cell cycle distribution was assessed using the ModFit LT software.

### Caspase assay

Cells were plated in 96-well plates and treated with JQ1 for 72 hours. Activities of caspase-3/7 were measured using a Caspase-Glo 3/7 Assay kit (Promega) in accordance to the manufacturer's instructions. Relative caspase 3/7 activities were calculated by normalizing the readings of caspase-3/7 activities to the readings of cell titers measured by a CellTiter-Glo Assay kit (Promega) in parallel wells.

### Colony formation assay

Ewing sarcoma cells were plated in 6-well plates in triplication and treated with DMSO or JQ1 the next day. Five days later, treatment was terminated. Cells were allowed to grow for seven to ten additional days prior to be fixed and stained with 0.5% crystal violet.

### Soft agar colony formation assay

Mouse NIH3T3 cells were infected with control lentivirus or lentivirus directing expression of EWS-FLI1. Cells were suspended in 0.35% low melting temperature agar (Sigma) with DMEM and plated on a 1% agar bottom layer. Cells were plated in triplicates at 10,000 cells per well in 6-well plates. Each well was topped with additional 2 mL of DMEM. On the second day, JQ1 was added to make the final concentrations 100 or 500 nmol/L. Cells were maintained in the presence of JQ1 for 5 days and cultured for 2 additional weeks after drug withdrawal before imaging.

### Subcutaneous xenograft assays

All animal experiments were performed under protocols approved by the Vanderbilt University Institutional Animal Care and Use Committee (IACUC). Female athymic nude mice 6–8 weeks old were used. TC32 or TC71 cells were trypsinized, suspended in PBS and mixed with equal volume of growth factor reduced Matrigel (BD Biosciences). Both flank sites of mice received injection of two million tumor cells. Tumor size were measured by a digital caliper and calculated following a formula of Size = Length × Width × Width/2. Treatment began when all tumors became palpable. Prior to treatment, animals were randomized into two groups (*n* = 8 for TC32 and *n* = 5 for TC71). However, a few mice bearing tumor significantly larger than others were assigned to the JQ1-treated arm. JQ1 was administrated via intraperitoneal injection at 50 mg/kg twice per day as described in our previous study [16]. Tumors were measured two to three times a week. Animals were monitored for significant adverse effects. Tumors were collected at the end of treatment and subjected to histological analysis by the Vanderbilt Translational Pathology Share Resource.

### Other statistical analyses

We employed GraphPad Prism 5.0 to determine statistical significance. Difference between two groups was determined by two-tailed unpaired Student's *t*-test. *P*-values of less than 0.05 were considered significant.

## SUPPLEMENTARY MATERIALS


