# Supplementary material for: BET bromodomain inhibitors suppress EWS-FLI1-dependent transcription and the IGF1 autocrine mechanism in Ewing sarcoma
Source: Oncotarget. 2016 Jun 1;7(28):43504–17. doi: 10.18632/oncotarget.9762 (PMC5190040; doi:10.18632/oncotarget.9762)
Supplement: Supplementary file 1 [file oncotarget-07-43504-s001.pdf]

BET bromodomain inhibitors suppress EWS-FLI1-dependent transcription and the IGF1 autocrine mechanism in Ewing sarcoma

Supplementary Materials

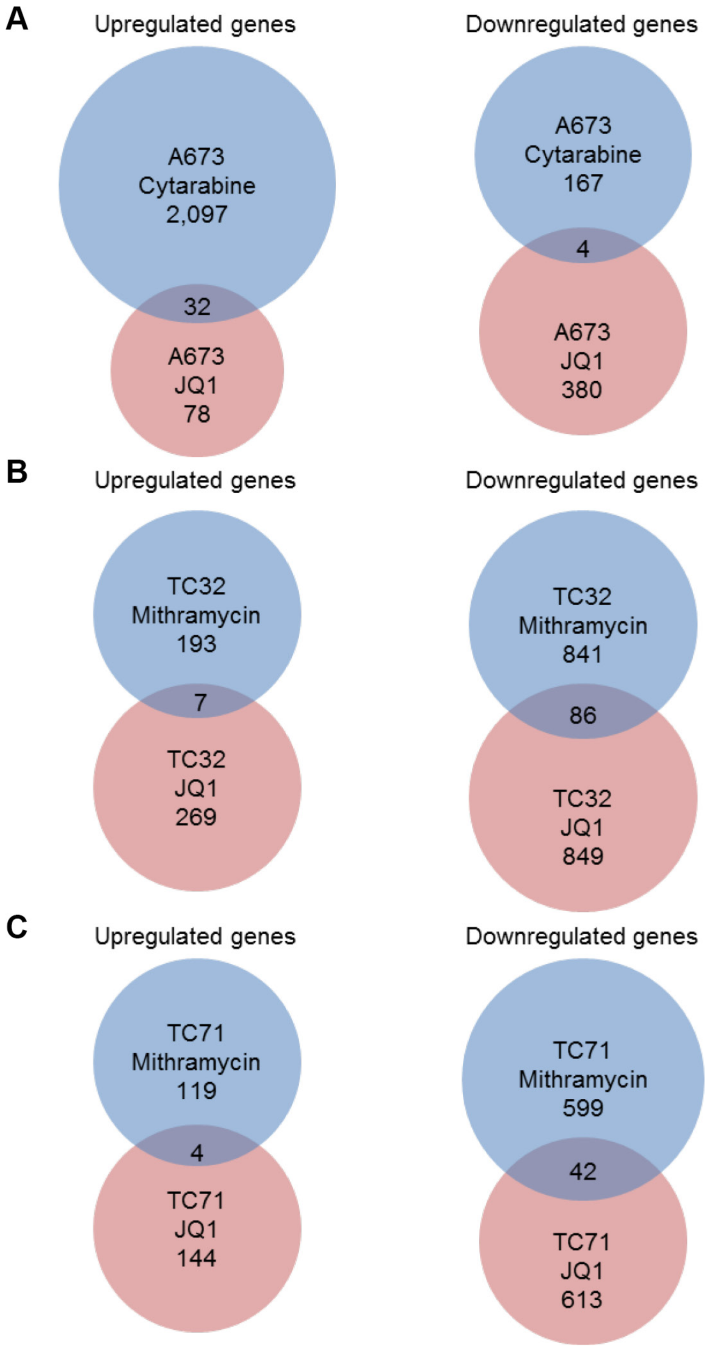

Supplementary Figure S1: Comparison of global gene expression following JQ1 treatment or chemotherapy drugs. (A–C) Venn diagrams summarize the genes that were differentially or commonly affected by JQ1, cytarabine (GEO: GSE6930) or mithramycin (GEO: GSE25127). Genes changed by more than 2 fold were selected for analyses.

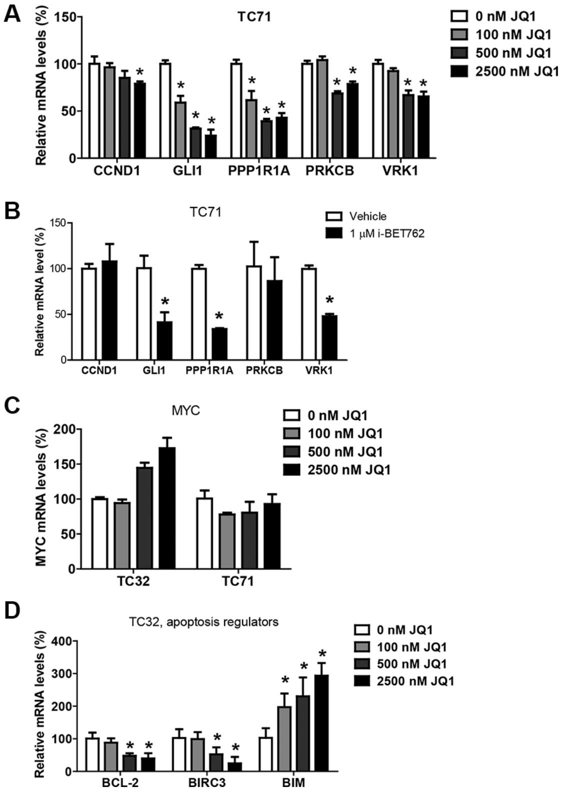

Supplementary Figure S2: BET inhibition represses expression of EWS-FLI1 target genes. (**A**) Expression of EWS-FLI1 target genes in TC71 cells treated with JQ1 at indicated concentrations or (**B**) 1 μmol/L i-BET762 for 24 hours, as assessed by qRT-PCR. (**C**) Expression of MYC in TC32 and TC71 cells treated as described above. (**D**) Expression of apoptotic regulators in TC32 cells treated as described above. $*p < 0.05$ by Student's *t*-test compared with control samples.

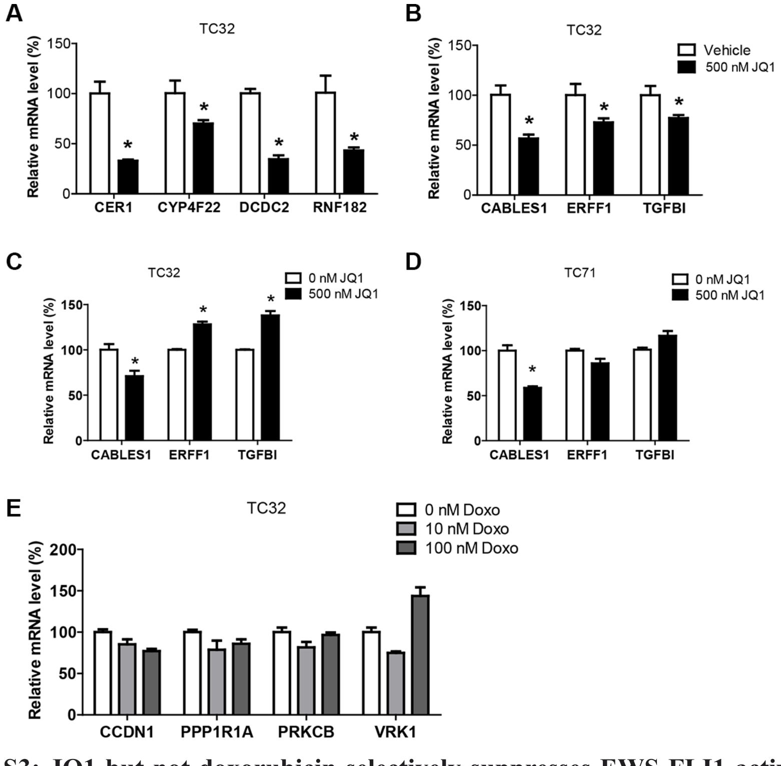

Supplementary Figure S3: JQ1 but not doxorubicin selectively suppresses EWS-FLI1-activated genes. (**A**) TC32 cells were treated with 500 nmol/L JQ1 for 4 hours. Expression of EWS-FLI1-activated and (**B**) repressed genes were determined by qRT-PCR. Actin was used as loading control. $*p < 0.05$ by Student's *t*-test compared with control samples. (**C**) TC32 or (**D**) TC71 cells were treated with 500 nmol/L JQ1 for 24 hours. Expression of selected genes repressed by EWS-FLI1 were determined by qRT-PCR. (**E**) TC32 cells were treated with doxorubicin at 10 or 100 nmol/L for 24 hours. Expression of selected EWS-FLI1 target genes were shown by qRT-PCR.

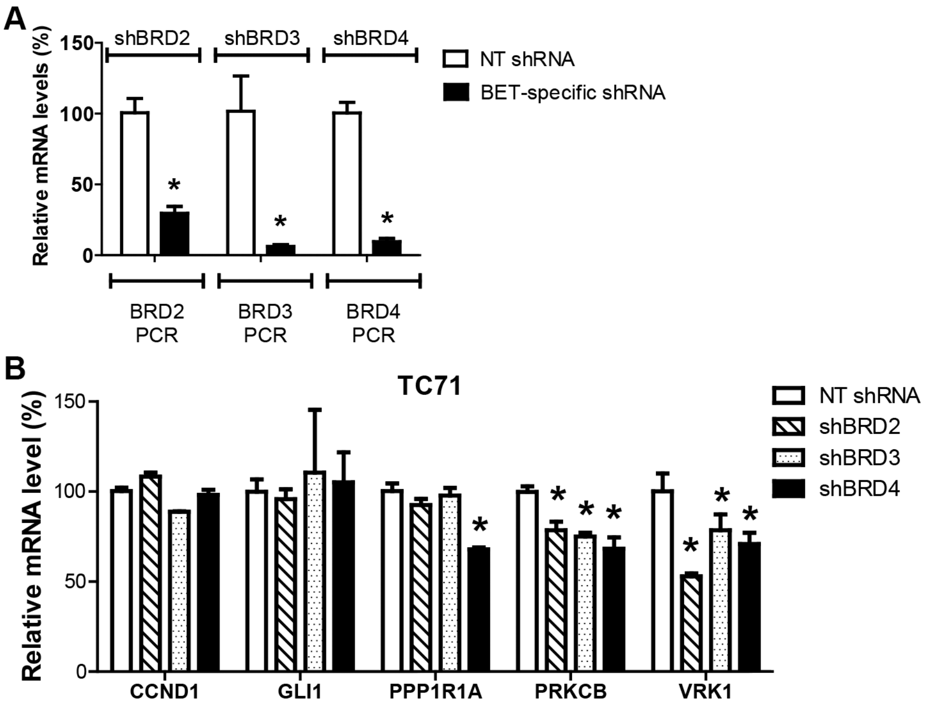

Supplementary Figure S4: Knockdown of BET genes downregulates EWS-FLI1 target genes. (**A**) Expression of BRD2, BRD3 and BRD4 in TC71 cells infected with lentivirus directing expression of shRNA sequences specific to BET genes, as measured by qRT-PCR. Knockdown efficiency in TC32 cells was similar (data not shown). (**B**) Expression of EWS-FLI1 in TC71 cells following knockdown of individual BET genes. *$p < 0.05$ by Student's t-test compared with control samples.

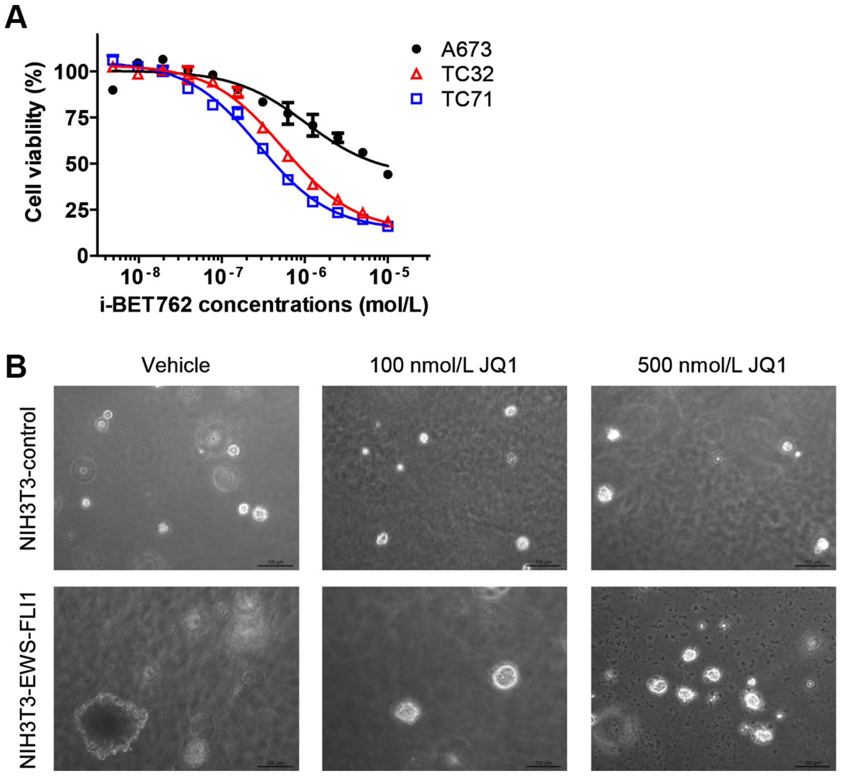

Supplementary Figure S5: BET inhibition impairs Ewing sarcoma cell viability and blocks NIH3T3 transformation. (**A**) Dose response curves of i-BET762 in Ewing sarcoma cell lines generated as described in Figure 5A. (**B**) NIH3T3 cells were infected with control lentivirus or lentivirus directing expression of EWS-FLI1. Following puromycin selection, cells were plated at 10,000 cells per well in 0.35% agar in 6-well plates. Cells were treated with 100 or 500 nmol/L JQ1 for 5 days and further incubated for 2 weeks in the absence of JQ1. Representative images are presented. Bars shown in the images represent 100 μm.

Supplementary Table S1: Sequences of qRT-PCR primers used in this study

Primer sequences		
ACTIN	forward	5′-TCGTGCGTGACATTAAGGA
	reverse	5′-AGGAAGGAAGGCTGGAAGAG
CCND1	forward	5′-CCGCTGGCCATGAACTACCT
	reverse	5′-ACGAAGGTCTGCGCGTGTT
GLI1	forward	5′-CTGGAGAACCTTAGGCTGGA
	reverse	5′-CGGCTGACTGTGTAAGCAGA
PPP1R1A	forward	5′-GCAACGGAAGAAGATGACAAG
	reverse	5′-AGGTTCCTCTCCTTGCTGCT
PRKCB	forward	5′-CTTCAAGCAGCCCACCTTCT
	reverse	5′-TCCCCGAAGCCCCAGATG
VRK1	forward	5′-CCAACGAGCTGCAAAACC
	reverse	5′-TGTCATGTAGACCAGACCCC
BCL2	forward	5′-ATGTGTGTGGAGAGCGTCAACC
	reverse	5′-TGAGCAGAGTCTTCAGAGACAGCC
BIRC3	forward	5′-TTGAACAGCTGCTATCCACATC
	reverse	5′-TCCAGGTTCAAAATGGATAATTG
BIM	forward	5′-CACAAACCCCAAGTCCTCCTT
	reverse	5′-TTCAGCCTGCCTCATGGAA
CER1	forward	5′-GAAATGCGGGTCTGTTCATT
	reverse	5′-CAGGCAAACAGTGAGAGCAG
CYP4F221	forward	5′-TTGGATGCTGTTCAATTTGG
	reverse	5′-GGCAGCTGAGTCAGATCGT
DCDC2	forward	5′-GAATTCACAAGAAACCATTCCAA
	reverse	5′-CCCCGTGTTTCAGACCTC
RNF182	forward	5′-CCGTTCTTAGAGGCAGGACTT
	reverse	5′-GGCCAGCTCTAGCAAACCTA
ERRFI1	forward	5′-ACCCACTGAAGTAGCTCATCG
	reverse	5′-TTCCATCTGAGTCTAACGTACCC
TGFBI	forward	5′-CGAGTGCTGTCCTGGATATG
	reverse	5′-CCCAGGGTCTCGTAAAGGTT
CABLES1	forward	5′-CACCGCAGCCTCTCCATA
	reverse	5′-GGGGTCATCCAAGAGATTTG
EWS-FLI1 primer set 1 [35]	forward	5′-TAGTTACCCACCCCAAACTGGAT
	reverse	5′-GGGCCGTTGCTCTGTATTCTTAC
EWS-FLI1 primer set 2 [36]	forward	5′-TCCTACAGCCAAGCTCCAAGTC
	reverse	5′-GAATTGCCACAGCTGGATCTGC
EWS-FLI1 primer set 3	forward	5′-CGACTAGTTATGATCAGAGCAGT
	reverse	5′-CCGTTGCTCTGTATTCTTACTGA
